# Association of socioeconomic status and overactive bladder in US adults: a cross-sectional analysis of nationally representative data

**DOI:** 10.3389/fpubh.2024.1345866

**Published:** 2024-03-26

**Authors:** Weilong Lin, Taibiao Li, Zhengyuan Xu, Peixin Chen, Qianqi Zheng, Ying-kai Hong, Wei-juan Liu

**Affiliations:** ^1^The First Affiliated Hospital of Shantou University Medical College, Medical College of Shantou University, Shantou, China; ^2^Department of Medical Cosmetic Center, the First Affiliated Hospital of Shantou University Medical College, Medical College of Shantou University, Shantou, China

**Keywords:** socioeconomic status, overactive bladder, poverty income ratio, NHANES, nocturia

## Abstract

**Background:**

Socioeconomic status inequality is an important variable in the emergence of urological diseases in humans. This study set out to investigate the association between the prevalence of overactive bladder (OAB) and the poverty income ratio (PIR) that served as a more influential indicator of socioeconomic status compared to education and occupation.

**Method:**

Data from the National Health and Nutrition Examination Survey (NHANES) conducted from 2007 to 2020 were used in this cross-sectional study. The association between the PIR and OAB was examined using weighted multivariate logistic regression and weighted restricted cubic splines (RCS). Additionally, interaction analysis was used for investigation to the connections between PIR and OAB in various covariate groups in order to confirm the stability of the results.

**Results:**

We observed a noteworthy inverse association between PIR and OAB after adjusting for potential confounding variables (OR = 0.87, 95% CI, 0.84–0.90, *p* < 0.0001). PIR was transformed into categorical variables, and the association held steady after that (1.0 < PIR <4.0 vs. PIR ≤ 1.0, OR = 0.70, 95% CI =0.63–0.77, *p* < 0.0001; PIR ≥ 4.0 vs. PIR ≤ 1.0, OR = 0.56, 95% CI =0.48–0.65, *p* < 0.0001). Additionally, RCS analysis showed that PIR and OAB had a negative nonlinear response relationship. Subgroup analyses showed that the inverse association between PIR and prevalence of OAB was stronger in obese than in nonobese individuals (P for interaction < 0.05).

**Conclusion:**

In our study, we observed a significant negative association between the PIR and the prevalence of OAB. In the future, PIR could be used as a reference standard to develop strategies to prevent and treat OAB.

## 1 Introduction

Socioeconomic status (SES) is a critical determinant of health in health-related studies, typically measured through education, occupation, and income (1). Nevertheless, models that employ education and occupational classifications have a tendency to ignore the individual’s capacity to acquire and express intentions to purchase health-related goods and services (2). The Poverty Income Ratio (PIR) metric has the potential to emerge as a more influential indicator of socioeconomic status compared to education and occupation. The PIR served as the official poverty measure used by the United States Census (3), compared to income, the use of PIR allows for the consideration of an appropriate poverty threshold that takes into account various family conditions and mitigates the impact of additional confounding factors. In the United States, income levels are strongly correlated with life expectancy, with higher incomes associated with longer lives, and the gap in life expectancy between income groups widened over time between 2001 and 2014 (4). A study in 2023 showed a large dose-dependent relationship between PIR and hypertension, diabetes, heart failure, coronary heart disease, stroke, all-cause mortality, and cardiac mortality (5). Further studies have confirmed the association of socioeconomic status (typically assessed by household income) with health conditions such as obesity, cancer and cardiovascular risk (6–8). However, to our best knowledge, the research on the association between Overactive Bladder (OAB) and socioeconomic status is scarce (6–8).

According to the International Continence Society (ICS), OAB was defined as a syndrome characterized by storage symptoms, particularly urgency, and may or may not include urgency urinary incontinence (UUI), often accompanied with increased daytime frequency of urination and nocturia (9). The prevalence of OAB in the United States is comparable between males (16.0%) and females (16.9%), and it tends to rise with increasing age (10). Among individuals under the age of 40 in six European countries, the prevalence of OAB was found to be 16.6%, and the urge incontinence is more frequently observed in females compared to males (11). OAB significantly affects the health-related quality of life and sleep of patients, leading to substantial negative consequences (12, 13), but its pathophysiology is still unclear (14). In the United States, individuals with OAB incur healthcare costs that exceed 2.5 times the expenses of those without OAB (15). OAB should be regarded as a complex symptom syndrome, arising from a multitude of potential mechanisms (14), while the relationship between PIR and OAB has not yet been explored for the USA population using nationally representative survey data. To address this research gap, broaden the research scope of OAB and enhance the overall effectiveness and efficiency of healthcare interventions for OAB, we utilized data from the National Health and Nutrition Examination Surveys (NHANES) conducted from 2007 to 2020, to explore the association between PIR and OAB.

## 2 Methods

### 2.1 Study settings

**Figure 1:**
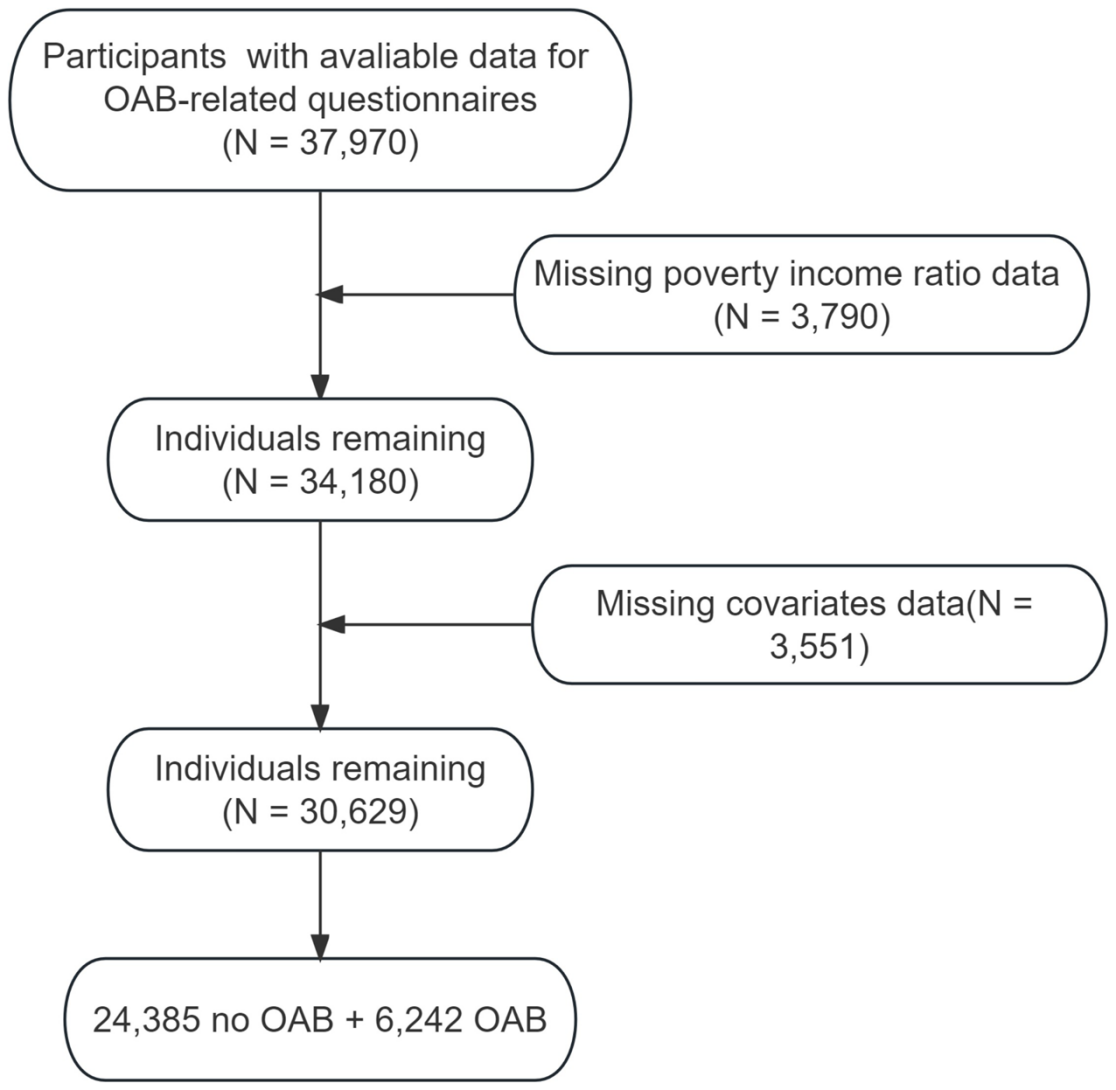
The selection process of NHANES 2007–2020.

We developed a cross-sectional study using data collected by the NHANES. The detailed information on NHANES design and methodology has been reported elsewhere.^1^ Briefly, NHANES, an ongoing program of cross-sectional studies approved by the National Center for Health Statistics aimed to assess the health and nutritional status of individuals residing in the United States. The survey uses a stratified multistage sampling design and combines data collected through interviews and physical examinations. For our analysis, we included participants of NHANES 2007–2020, aged over 20 years old who had available information for OAB-related questionnaires. Participants with missing data on outcome, covariates or poverty income ratio information were excluded (Figure 1).

### 2.2 Study variables

The definition of OAB includes the presence of urge urinary incontinence and nocturia in a patient. Previous research supports the efficacy of overactive bladder symptom score (OABSS), which has been further utilized to further quantify the symptoms of OAB in order to improve diagnosis (16, 17). We used specific criteria to diagnose OAB, when the total score of OABSS is ≥3, the diagnosis is OAB. Supplementary Table S1 for specific scoring criteria.

We utilize the PIR, which is a ratio of family income to the poverty threshold (3). And the calculation of PIR involves dividing the reported total family income by the poverty level established by the Department of Health and Human Services (HHS) (18). If the family income was reported as a range, the PIR was calculated by taking the midpoint of the reported range. A low PIR indicates a greater degree of poverty. A PIR of 1.0 (income at 100% of the poverty level) indicates that an individual is living at the poverty line. Meanwhile, a ratio of 0.5 (income at 50% of the poverty level) would signify that one resides in a household earning just half of the income specified as the poverty threshold. To categorize them accurately, previous research suggested dividing the PIR into three groups: low-income level (PIR ≤ 1), middle-income level (1 < PIR < 4), and high-income level (PIR ≥ 4) (19).

Based on numerous studies of risk factors for OAB, we incorporated the following variables as covariates: age, gender, ethnicity, marital status, level of education, Body Mass Index (BMI), recreational activities, smoking and alcohol consumption habits, hypertension, diabetes, cardiovascular diseases (CVD), depression, cancer, and urinary creatinine (20–25). All covariates in our analysis can be found on the official NHANES website.^2^

For the categorization of covariates, race groups were classified as follows: White (non-Hispanic White), Black (non-Hispanic Black), Hispanic (Hispanic American), and Other (other race including multi-racial and other Hispanic). Education levels were categorized as: “less than high school” (9th–11th grade and 12th grade with no diploma), “high school or equivalent” (high school graduate/GED or equivalent), and “college or above” (some college or AA degree). Smoking status was classified into the following categories: “never smoked” (less than 100 cigarettes in a lifetime), “former smokers” (more than 100 cigarettes in a lifetime but currently not smoking), and “current smokers” (individuals who are currently smoking). Similarly, drinking status was divided into “never drinking” (less than 12 drinks in a lifetime), “former drinkers” (more than 12 drinks in a lifetime but did not drink last year), and “current drinkers” (more than 12 drinks in a lifetime and still drinking recently). Hypertension was diagnosed based on the following criteria: (1) systolic and diastolic blood pressure measurements on two different occasions, with the mean of these measurements (≥140/90 mmHg), (2) confirmation of hypertension by a physician’s diagnosis, and (3) self-reported use of antihypertensive medications. The diagnosis of diabetes mellitus is established if any of the following criteria are met: (1) Your doctor has confirmed that you have diabetes, (2) Glycosylated hemoglobin (HbA1c) (%) > 6.5, (3) fasting blood glucose (mmol/L) ≥ 7.0, (4) random blood glucose (mmol/L) ≥ 11.1, (5) 2-h OGTT blood glucose (mmol/L) ≥ 11.1, and (6) taking diabetes medication or insulin. Cardiovascular disease (CVD) was defined as a self-reported medical history of one of the following conditions: coronary heart disease, myocardial infarction, congestive heart failure, or stroke. The assessment of depression was conducted using the Patient Health Questionnaire-9 (PHQ-9), a widely utilized instrument for evaluating the severity of depressive symptoms, with depression defined as a total score ≥ 10. The presence of cancer was evaluated through a questionnaire in the study. In contrast to the original NHANES dataset, modifications were made to the educational level variable by recoding it, and apart from that some race groups and the marital status variable was integrated.

### 2.3 Statistical analysis

In order to consider the impacts of the intricate multistage sampling design of NHANCES, the utilization of appropriate sample weights was employed to enhance data accuracy, adhering to NHANES guidelines. In this study, statistical analyses were performed using both R software version 4.2.3 and the nhanesR software package. The continuous variables in the baseline data are represented by weighted mean and standard error. Categorical variables are expressed as weighted percentages and frequencies. Depending on the distribution, differences between continuous variables are assessed using Analysis of variance (ANOVA) tests or Kruskal-Wallis tests. Chi-square tests were used to analyze differences between categorical variables. Calculate standard errors using the Taylor method. Weight logistic regression analysis were used to investigate the relations between OAB and PIR when adjusted for covariates. In addition, we performed logistic regression analysis of PIR and OAB by gender separately, and also carried sensitivity analysis of odds of OAB after adjusting for delivery. We constructed 4 logistic regression models: model 1 adjusted for no variables; model 2 adjusted for age, sex, and race; model 3 adjusted for age, sex, race, marital status, education level, BMI, recreational activity, smoking status, and drinking status; model 4 further adjusted for creatinine urine, hypertension, diabetes, CVD, depression as well as cancer. What’s more, dose–response relationship analysis was performed. In model 4, weighted restricted cubic splines (RCS) with three knots were employed to achieve a flexible and precise depiction of the association between PIR and OAB. The subgroup analyses and multiplicative interaction tests were carried out by dividing the participants into different subgroups based on specific characteristics such as age group, race, smoking/drinking status and potential confounding factors. These subgroups were then analyzed separately to explore the relationships between PIR and OAB within different groups of condition covariates. To account for the potential impact of excluding a significant number of participants due to missing data, we employed multiple imputation, a sensitivity analysis for the logistic regression analysis (all missing values for the continuous covariates had been imputed). In this study, survey package was used for logistic regression, rcs package for RCS curves, and mice package for multiple imputation. Two-tailed *p* value <0.05 was considered statistically significant.

### 2.4 Ethical considerations

The National Center for Health Statistics Research Ethics Review Board (National Center for Health Statistics, 2012) obtained the ethics approval for the NHANES study. Written consent was obtained from all NHANES participants during the recruitment process.

## 3 Results

### 3.1 The selection of NHANES (2007–2020)

Initially, we incorporated data pertaining to a total of 37,970 participants who completed OAB-related questionnaires in our study. After excluding the data from 3,790 participants missing poverty income ratio information and 3,551 participants without necessary covariates, our final sample data came from 30,629 participants, of which 6,242 were classified as having OAB and 24,385 were classified as not having OAB. For visual reference, please refer to Supplementary Figure S1, which presents a participant flow chart.

### 3.2 Demographic characteristics

The research encompassed 30,629 American individuals, whereby 50.95% were female, 49.05% were male, and 15.54% were diagnosed with OAB. PIR-dependent participant characteristics can be found in Table 1. For each PIR classification, the incidence of OAB was 22.22, 17.00, and 11.45% among low, middle and high-income groups, correspondingly. Additionally, individuals with lower income had lower education levels, were less physically active, exhibited higher smoking rates, and had a greater propensity to develop diabetes, cardiovascular disease, depression, and cancer relative to higher-income respondents. Table 1 presents detailed demographic data for all respondents.

**Table 1:** Demographic characteristics stratified by Trichotomous of PIR (*N* = 30, 629).

Characteristic	Poverty income ratio				
	Total	≤1.0 (*N* = 6,322)	1.0–4.0 (*N* = 16,128)	≥4.0 (*N* = 8,179)	*p*-value
	30,629	(*N* = 6,322)	(*N* = 16,128)	(*N* = 8,179)	
		(20.64%)	(52.66%)	(26.70%)	
Sex					<0.0001
Male	15,187 (49.05)	2,905 (45.30)	8,016 (48.16)	4,266 (51.45)	
Female	15,442 (50.95)	3,417 (54.70)	8,112 (51.84)	3,913 (48.55)	
Age*					<0.0001
<50	15,540 (55.54)	3,617 (66.93)	8,083 (56.96)	3,840 (49.87)	
≥50	15,089 (44.46)	2,705 (33.07)	8,045 (43.04)	4,339 (50.13)	
Race					<0.0001
White	12,901 (67.86)	1,973 (45.77)	6,648 (64.07)	4,280 (80.16)	
Black	6,620 (10.52)	1,628 (18.62)	3,658 (12.16)	1,334 (5.70)	
Hispanic	4,307 (8.10)	1,260 (16.24)	2,470 (9.81)	577 (3.17)	
Other	6,801 (13.52)	1,461 (19.37)	3,352 (13.96)	1,988 (10.97)	
BMI*					<0.0001
<30.00	18,490 (60.76)	3,758 (60.13)	9,487 (58.50)	5,245 (63.74)	
≥30.00	12,139 (39.24)	2,564 (39.87)	6,641 (41.50)	2,934 (36.26)	
Marital status					<0.0001
Married/living with partner	18,223 (63.50)	2,845 (45.10)	9,430 (59.11)	5,948 (75.26)	
single/divorced/widowed	12,406 (36.50)	3,477 (54.90)	6,698 (40.89)	2,231 (24.74)	
Education level					<0.0001
Less than high school	6,542 (12.95)	2,546 (32.85)	3,589 (15.24)	407 (3.25)	
High school or equivalent	6,971 (23.43)	1,673 (30.14)	4,196 (28.61)	1,102 (14.76)	
College or above	17,116 (63.62)	2,103 (37.02)	8,343 (56.15)	6,670 (82.00)	
Recreational activity					<0.0001
No activity	15,519 (43.97)	3,910 (57.89)	8,684 (49.37)	2,925 (32.52)	
Moderate	7,948 (27.94)	1,300 (20.72)	4,160 (26.88)	2,488 (31.74)	
Vigorous	7,162 (28.09)	1,112 (21.38)	3,284 (23.74)	2,766 (35.74)	
Smoking status					<0.0001
Never	17,044 (56.38)	3,059 (47.87)	8,815 (53.88)	5,170 (62.40)	
Former	7,344 (24.85)	1,161 (16.60)	4,004 (24.91)	2,179 (27.63)	
Now	6,241 (18.77)	2,102 (35.53)	3,309 (21.21)	830 (9.97)	
Drinking status					<0.0001
No	4,029 (9.62)	1,142 (15.59)	2,193 (11.11)	694 (5.72)	
Former	3,951 (9.36)	1,019 (12.69)	2,280 (10.98)	652 (6.24)	
Now	22,649 (81.02)	4,161 (71.72)	11,655 (77.91)	6,833 (88.05)	
Hypertension					0.01
No	17,943 (63.61)	3,696 (64.72)	9,256 (62.16)	4,991 (65.01)	
Yes	12,686 (36.39)	2,626 (35.28)	6,872 (37.84)	3,188 (34.99)	
Diabetes					<0.0001
No	24,895 (85.86)	5,027 (84.43)	12,929 (84.73)	6,939 (87.75)	
Yes	5,734 (14.14)	1,295 (15.57)	3,199 (15.27)	1,240 (12.25)	
CVD					<0.0001
No	27,439 (91.83)	5,575 (90.38)	14,275 (90.54)	7,589 (93.91)	
Yes	3,190 (8.17)	747 (9.62)	1,853 (9.46)	590 (6.09)	
Depression					<0.0001
No	27,903 (92.08)	5,306 (83.16)	14,732 (91.47)	7,865 (95.92)	
Yes	2,726 (7.92)	1,016 (16.84)	1,396 (8.53)	314 (4.08)	
Cancer					<0.0001
No	27,681 (89.77)	5,904 (93.78)	14,553 (90.46)	7,224 (87.54)	
Yes	2,948 (10.23)	418 (6.22)	1,575 (9.54)	955 (12.46)	
OAB					<0.0001
No	24,387 (84.46)	4,660 (77.78)	12,694 (83.00)	7,033 (88.55)	
Yes	6,242 (15.54)	1,662 (22.22)	3,434 (17.00)	1,146 (11.45)	
Creatinine urine (mg/dl)*	121.58 (0.85)	130.19 (1.96)	124.93 (1.12)	114.49 (1.34)	<0.0001

Analysis of variance (ANOVA) tests or Kruskal-Wallis tests were used to analyze differences between continuous variables (marked with *).

Chi-square tests were used to analyze differences between categorical variables.

PIR, poverty income ratio; BMI, body mass index; CVD, cardiovascular diseases; OAB, overactive bladder.

### 3.3 Associations between PIR and OAB

The findings of a weighted multivariate logistic regression analysis are presented in Table 2, revealing an inverse association between PIR and the prevalence of OAB. The statistical association were significant across models 1 (OR = 0.82; 95%CI = 0.80–0.84, *p* < 0.0001), model 2 (OR = 0.79; 95%CI = 0.77–0.81, *p* < 0.0001), and model 3 (OR = 0.85; 95%CI = 0.83–0.88, *p* < 0.0001). Furthermore, in model 4, the observed results continued to be statistically significant after adjusting for all covariates (OR = 0.87; 95%CI = 0.84–0.90, *p* < 0.0001).

**Table 2:** Logistic regression analysis of PIR and overactive bladder.

	Model 1	*p*-value	Model 2	*p*-value	Model 3	*p*-value	Model 4	*p*-value
	OR (95%CI)		OR (95%CI)		OR (95%CI)		OR (95%CI)	
PIR	0.82 (0.80, 0.84)	<0.0001	0.79 (0.77, 0.81)	<0.0001	0.85 (0.83, 0.88)	<0.0001	0.87 (0.84, 0.90)	<0.0001
Classification of PIR								
≤1.0	1		1		1		1	
1.0–4.0	0.72 (0.65, 0.79)	<0.0001	0.56 (0.50, 0.62)	<0.0001	0.65 (0.59, 0.72)	<0.0001	0.70 (0.63, 0.77)	<0.0001
≥4.0	0.45 (0.40, 0.52)	<0.0001	0.35 (0.31, 0.41)	<0.0001	0.51 (0.44, 0.60)	<0.0001	0.56 (0.48, 0.65)	<0.0001
P for trend		<0.0001		<0.0001		<0.0001		<0.0001

Model 1: unadjusted.

Model 2: adjusted for age, sex, and race.

Model 3: adjusted for age, sex, race, marital status, education level, BMI, recreational activity, smoking status, and drinking status.

Model 4: further adjusted for creatinine urine, hypertension, diabetes, CVD, depression as well as cancer.

Meanwhile, in order to conduct further analysis, PIR was transformed from a continuous variable to a categorical variable. The findings illustrated that when compared to the low-income group, the probability of OAB for the high-income group was 45, 35, 51, and 56% in models 1, 2, 3, and 4, respectively (All *p* < 0.0001) (Table 2).

To eliminate the impact of gender on the results, we segregated the gender-specific samples for further examination, the results showed that the prevalence of OAB was significantly inversely associated with PIR in both men and women (Supplementary Table S2).

Vaginal delivery has been identified as a notable risk factor for the development of postpartum urinary incontinence (26). We stratified the delivery condition based on the number of vaginal deliveries into four distinct levels (0 times, 1–2 times, 3–4 times, ≥5 times) and a sensitivity analysis was conducted which yielded no change in the primary outcome when the delivery in women were taken as confounding factors (OR = 0.88, 95% CI = 0.83–0.92, *p* < 0.0001) (Supplementary Table S3).

**Figure 2:**
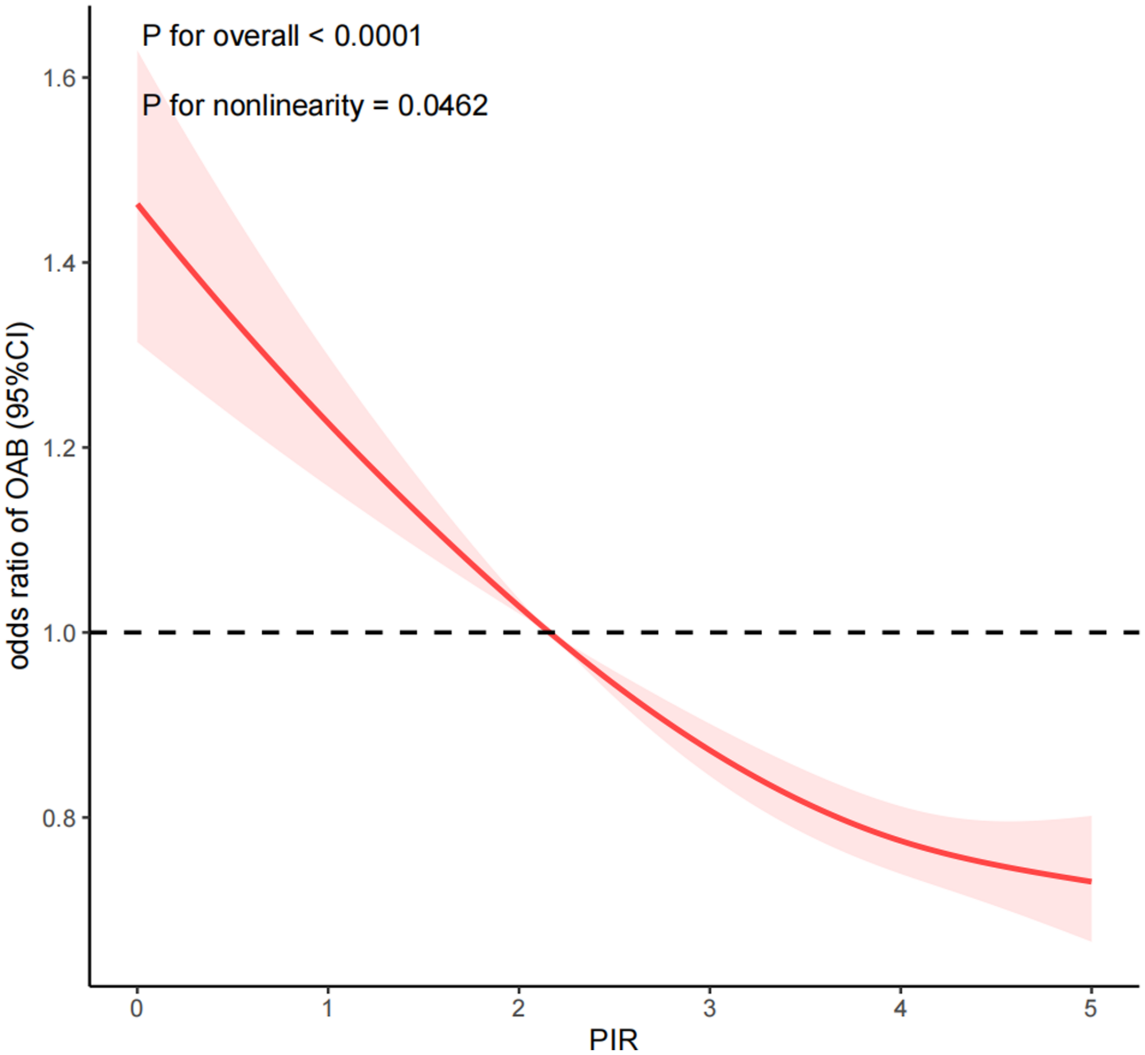
Dose-response relationship analysis between PIR and OAB. PIR: poverty income ratio, OAB: overactive bladder. RCS regression was adjusted for age, sex, race, marital status, education level, BMI, recreational activity, smoking status, drinking status, creatinine urine, hypertension, diabetes, CVD, depression, and cancer (Model 4). The red solid line represents ORs, red shaded region represents 95% CI.

Furthermore, the dose–response association between PIR and OAB was analyzed, revealing a negative nonlinear relationship between the two (P for overall < 0.0001; P for nonlinearity = 0.0462) as shown in Figure 2. This suggests that an increase in PIR corresponds to a decrease in the prevalence of developing OAB. In sex-specific dose–response analyses, a linear inverse association was observed between PIR and prevalence of OAB in men (P for overall < 0.0001, P for nonlinearity = 0.8996). In the female population, PIR was negatively correlated with the prevalence of OAB (P for overall <0.0001, P for nonlinearity = 0.0078). Of note, the piecewise regression indicated a dose–response curve cut point of 4.824 for PIR and prevalence of OAB in the female population. This indicates that when PIR is less than 4.824, the prevalence of OAB decreases significantly as PIR increases. When PIR exceeded 4.824, the prevalence of OAB decreased gradually. However, when PIR > 4.824, with the increase of PIR, the prevalence reduction trend of OAB tended to be flat (Supplementary Figure S1).

**Figure 3:**
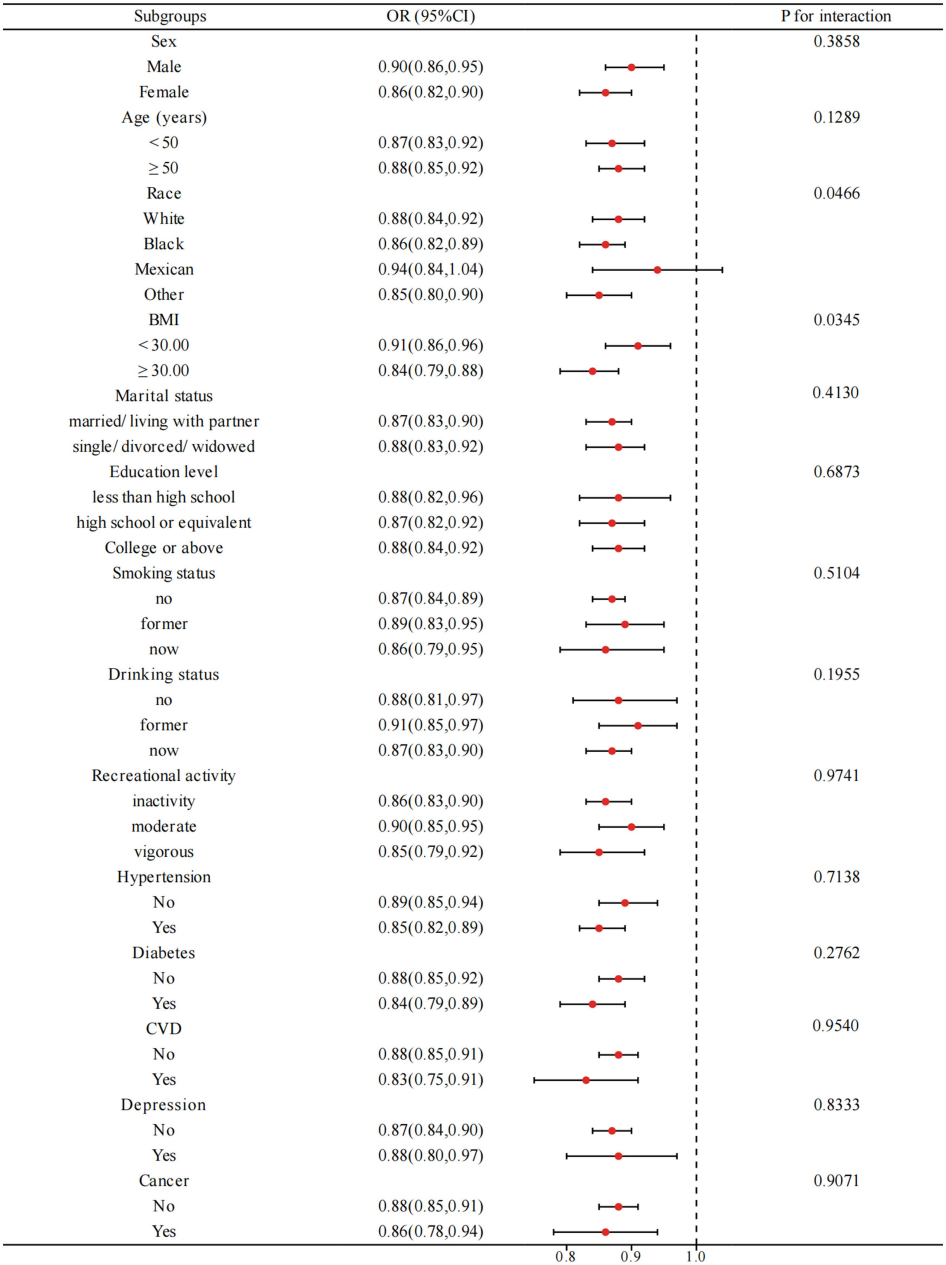
Subgroup analysis for the relationship between PIR and OAB. BMI: body mass index, CVD: cardiovascular diseases. Analyses were adjusted for age, sex, race, marital status, education level, BMI, recreational activity, smoking status, drinking status, creatinine urine, hypertension, diabetes, CVD, depression, and cancer.

Figure 3 illustrates the outcome of subgroup analyses, wherein age, race, BMI, smoking status, drinking status, recreational activity, hypertension, diabetes, cardiovascular disease, and depression were considered. Our observations demonstrate that PIR and OAB are negatively correlated across all subgroups. Interaction testing indicated that BMI significantly altered the association between PIR and OAB (P for interaction < 0.05), but the remaining subgroups had no considerable effect on this negative association.

In order to improve the sensitivity of the results, we implemented multiple imputation techniques to address the potential bias resulting from the exclusion of a considerable number of participants due to missing data. And the result remained stable through the utilization of a multiple imputation technique evidenced by the comparison to Table 2 (Supplementary Table S4).

## 4 Discussion

### 4.1 Reliability of result

In this study conducted on a representative sample of the adult United States population, we observed a significant association between PIR and OAB. Both univariate and multivariate logistic regression analyses demonstrated that the level of PIR was associated with a reduced prevalence of OAB. Transforming PIR from a continuous variable to a categorical variable revealed a significantly reduced prevalence of OAB in the higher income group compared to the lower income group. Further analysis of the dose–response relationship showed a nonlinear inverse association between PIR and prevalence of OAB. Subgroup analysis showed that the inverse association between PIR and prevalence of OAB was stronger in obese people than in non-obese people, while the stability of the association between PIR and OAB prevalence remained consistent in other subgroups.

Many previous epidemiological studies have demonstrated that socioeconomic disparities in wealth have a significant impact on individuals’ overall health (1, 27). It appears to be a complex association between income, obesogenic behaviors, prevalence of obesity, adiposity level and cardiovascular disease (6, 8). What’s more, there was a large dose-dependent association of PIR with the prevalence of hypertension, diabetes, CHF, CAD, stroke, all-cause mortality, and cardiac mortality (5). A multi-cohort study demonstrated a positive association between low socioeconomic status and an elevated risk for multiple diseases in comparison to more advantaged individuals (28). Low levels of PIR accompanied by poor health conditions were likely to induce OAB.

Findings of other studies indicated that the above risk factors related to PIR, such as obesity, abnormal metabolic factors, age, diabetic duration and so on are intimately associated with OAB (20, 21, 23–25). To eliminate the impact of any extraneous variables, we created four models that accounted for various covariates of OAB. Through logistic regression analysis, we were able to confirm a consistently significant association between PIR and OAB even after adjusting for these factors.

### 4.2 Strengths and weaknesses

There may be other potential mediating factors between PIR and OAB. PIR should represent various states of health to some extent influence the prevalence of OAB. Our findings and conclusions reinforce this. The income levels influence the choice of treatment for OAB patients (29), and the low use of advanced treatments is partly related to the high healthcare costs of OAB patients. In this case, PIR further affects the course prognosis of OAB. In addition, socioeconomic status (SES) significantly impacts the incidence and quality of life for individuals with neurologic and developmental disorders in childhood that low-income groups live with increased prevalence of many neurological diseases, while groups with high income adopt healthy but expensive lifestyles early in life (30, 31).

OAB should be acknowledged as a complex symptom syndrome, influenced by various potential mechanisms, making it a multifactorial condition (14). It is difficult to explain all the factors associated with OAB due to the complex interactions between multiple known and unknown disease factors, environmental factors, economic factors, and genetic factors. Therefore, it is worth investigating the prevalence and characteristics of OAB to identify potential evidence for enhancing strategies to decrease its morbidity by reviewing studies on the comorbidity of OAB. In this study, PIR serves as a proxy for health status groups that are considered to have comorbidities related to OAB. The accessible PIR could be applied to strategies for the management of OAB after the validation process. For instance, the evaluation of the PIR during a health examination has the potential to facilitate the screening of OAB and the implementation of relevant preventive measures. Moreover, in the realm of OAB diagnosis and treatment, the influence of the PIR level can be taken into account and specifically addressed to enhance treatment outcomes.

This cross-sectional study utilizes the NHANES database to explore the association between PIR and the prevalence of overactive bladder. However, some limitations should be acknowledged. Firstly, the nature of this cross-sectional study raises questions regarding the involvement of causality or temporal variation. Secondly, unstable information on overactive bladder is caused by using the OABSS scale and NHANES. The questionnaire informed the OABSS scale in the NHANES database, but the responsiveness of OABSS to change remains uncertain that it is not reliable enough for assessing OAB severity (16). The presence of different severity levels of OAB may act as a confounding factor in the association between PIR and OAB. Thirdly, the use of OR as an effect measure in cross-sectional studies when the outcome is not incidence of disease can lead to overestimation of magnitude of associations. Nonetheless, our study benefits from utilizing data from an extensive and representative cross-sectional survey (NHANES). Most importantly, the study presents findings suggesting a substantial association between PIR and OAB, which has not been previously identified. This research is expected to significantly contribute to the management and treatment of OAB.

## 5 Conclusion

Our research indicated that individuals in the United States with higher PIR were less likely to have OAB. The PIR should be considered in the development of strategies aimed at managing OAB. Additional studies are required to investigate its underlying mechanism in the future.

## Data availability statement

Publicly available datasets were analyzed in this study. This data can be found here: https://www.cdc.gov/nchs/nhanes/index.htm.

## Ethics statement

The studies involving humans were approved by The National Center for Health Statistics Research Committee. The studies were conducted in accordance with the local legislation and institutional requirements. The participants provided their written informed consent to participate in this study.

## Author contributions

WL: Writing – review & editing, Writing – original draft, Validation, Supervision, Formal analysis, Data curation, Conceptualization. TL: Writing – review & editing, Writing – original draft, Methodology, Formal analysis, Data curation. ZX: Writing – original draft, Methodology, Formal analysis, Conceptualization. PC: Writing – original draft, Data curation. Y-kH: Writing – review & editing, Writing – original draft, Validation, Supervision, Formal analysis, Data curation. W-jL: Writing – review & editing, Writing – original draft, Validation, Supervision, Formal analysis, Data curation. QZ: Writing – original draft, Methodology.

## Footnotes

^1^ http://www.cdc.gov/nchs/nhanes/index.htm

^2^ https://www.cdc.gov/nchs/nhanes/
