# Supplementary material for: Association of socioeconomic status and overactive bladder in US adults: a cross-sectional analysis of nationally representative data
Source: Front Public Health. 2024 Mar 26;12:1345866. doi: 10.3389/fpubh.2024.1345866 (PMC11003547; doi:10.3389/fpubh.2024.1345866)
Supplement: Supplementary file 1 [file Data_Sheet_1.docx]

**Supplementary Table 1** **Criteria for Conversion of Symptom Frequencies recorded in NHANES and OABSS Scores**

| **According to NHANES Score** | **According to OABSS Score** |
| --- | --- |
| Urge urinary incontinence frequency | Urge urinary incontinence score |
| Never | 0 |
| Less than once a month | 1 |
| A few times a month | 1 |
| A few times a week | 2 |
| Every day or night | 3 |
| Nocturia frequency | Nocturia score |
| 0 | 0 |
| 1 | 1 |
| 2 | 2 |
| 3 | 3 |
| 4 | 3 |
| 5 or more | 3 |
| When total score ≥3, the diagnosis is OAB | |

NHANES = National Health and Nutrition Examination Survey; OABSS = Overactive Bladder Symptom Score

**Supplementary Table 2 Logistic regression analysis of PIR and Overactive bladder by gender**

|  | Model 1 | P-value | Model 2 | P-value | Model 3 | P-value | Model 4 | P-value |
| --- | --- | --- | --- | --- | --- | --- | --- | --- |
|  | OR (95%CI) | | OR (95%CI) | | OR (95%CI) | | OR (95%CI) | |
| Male | | | | | | | | |
| PIR | 0.86(0.82,0.90) | <0.0001 | 0.81(0.77,0.85) | <0.0001 | 0.88(0.84,0.93) | <0.0001 | 0.90(0.86,0.95) | <0.001 |
| Classification of PIR | | | | | | | | |
| ≤ 1.0 | 1 |  | 1 |  | 1 |  | 1 |  |
| 1.0 - 4.0 | 0.73(0.62,0.86) | <0.001 | 0.54(0.46,0.64) | <0.0001 | 0.63(0.54,0.74) | <0.0001 | 0.69(0.59,0.81) | <0.0001 |
| ≥ 4.0 | 0.51(0.41,0.65) | <0.0001 | 0.36(0.29,0.45) | <0.0001 | 0.54(0.44,0.67) | <0.0001 | 0.60(0.48,0.75) | <0.0001 |
| P for trend | | <0.0001 |  | <0.0001 |  | <0.0001 |  | <0.0001 |
| Female | | | | | | | | |
| PIR | 0.80(0.78,0.83) | <0.0001 | 0.78(0.75,0.81) | <0.0001 | 0.84(0.80,0.88) | <0.0001 | 0.86(0.82,0.90) | <0.0001 |
| Classification of PIR | | | | | | | | |
| ≤ 1.0 | 1 |  | 1 |  | 1 |  | 1 |  |
| 1.0 - 4.0 | 0.72(0.63,0.83) | <0.0001 | 0.57(0.50,0.65) | <0.0001 | 0.68(0.59,0.78) | <0.0001 | 0.73(0.63,0.83) | <0.0001 |
| ≥ 4.0 | 0.43(0.37,0.50) | <0.0001 | 0.35(0.29,0.41) | <0.0001 | 0.51(0.42,0.62) | <0.0001 | 0.57(0.47,0.69) | <0.0001 |
| P for trend | | <0.0001 |  | <0.0001 |  | <0.0001 |  | <0.0001 |

Model1: unadjusted.

Model2: adjusted for age and race

Model3: adjusted for age, race, marital status, education level, BMI, recreational activity, smoking status, and drinking status.

Model4: further adjusted for creatinine urine, hypertension, diabetes, CVD, depression as well as cancer.

**Supplementary Table 3 Sensitivity analysis of odds of OAB after separately adjusting for delivery**

|  | OR (95CI%) | P-value |
| --- | --- | --- |
| PIR | 0.88(0.83,0.92) | <0.0001 |
| Classification of PIR | | |
| ≤ 1.0 | 1 |  |
| 1.0 - 4.0 | 0.75(0.65,0.86) | <0.001 |
| ≥ 4.0 | 0.61(0.49,0.75) | <0.0001 |
| P for trend |  | <0.0001 |

Analyses were adjusted for age, race, marital status, education level, PIR, recreational activity, smoking status, and drinking status, creatinine urine, hypertension, diabetes, CVD, depression, cancer, and number of vaginal deliveries.

**Supplementary Ta****ble 4 Multiple Imputation for** **Logistic regression analysis of PIR and Overactive bladder**

|  | Model 1 | P-value | Model 2 | P-value | Model 3 | P-value | Model 4 | P-value |
| --- | --- | --- | --- | --- | --- | --- | --- | --- |
|  | OR (95%CI) | | OR (95%CI) | | OR (95%CI) | | OR (95%CI) | |
| PIR | 0.81(0.79,0.83) | <0.0001 | 0.79(0.76,0.81) | <0.0001 | 0.86(0.83,0.89) | <0.0001 | 0.88(0.85,0.91) | <0.0001 |
| Classification of PIR | | | | | | | | |
| ≤ 1.0 | 1 |  | 1 |  | 1 |  | 1 |  |
| 1.0 - 4.0 | 0.72(0.65,0.80) | <0.0001 | 0.55(0.50,0.62) | <0.0001 | 0.66(0.60,0.73) | <0.0001 | 0.70(0.64,0.78) | <0.0001 |
| ≥ 4.0 | 0.43(0.38,0.49) | <0.0001 | 0.34(0.30,0.40) | <0.0001 | 0.52(0.46,0.60) | <0.0001 | 0.58(0.50,0.67) | <0.0001 |
| P for trend | | <0.0001 |  | <0.0001 |  | <0.0001 |  | <0.0001 |

All missing values for the continuous covariates were imputed.

Model1: unadjusted.

Model2: adjusted for age, sex, and race.

Model3: adjusted for age, sex, race, marital status, education level, BMI, recreational activity, smoking status, and drinking status.

Model4: further adjusted for creatinine urine, hypertension, diabetes, CVD, depression as well as cancer.

**Supplementary Figure 1 Dose-response relationship analysis between PIR and OAB in different sex groups**

PIR: poverty income ratio, OAB: overactive bladder

RCS regression was adjusted for age, race, marital status, education level, BMI, recreational activity, smoking status, drinking status, creatinine urine, hypertension, diabetes, CVD, depression, and cancer. The red and blue solid lines represents ORs, red and blue shaded regions represents 95 % CI.

**
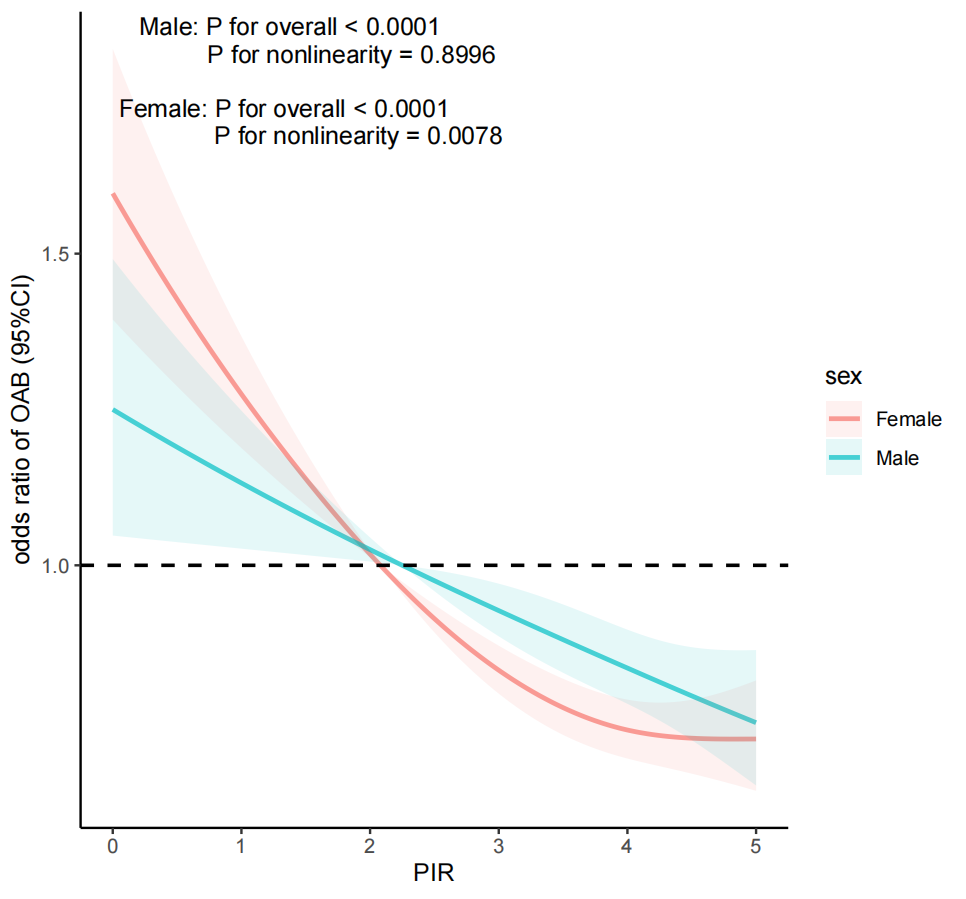
**
